# TSPO in systemic-ocular immunometabolic crosstalk: mechanisms and therapeutic implications

**DOI:** 10.3389/fimmu.2026.1929816

**Published:** 2026-10-06

**Authors:** Zuyi Yang, Xinyi Zhang, Dianzhe Tian, Xinshi Li, Xinyu Zhao, Erqian Wang, Youxin Chen

**Affiliations:** 1 Department of Ophthalmology, Key Laboratory of Ocular Fundus Diseases, Peking Union Medical College Hospital, Chinese Academy of Medical Sciences and Peking Union Medical College, Beijing, China; 2 Department of Liver Surgery, State Key Laboratory of Complex Severe and Rare Diseases, Peking Union Medical College Hospital, Chinese Academy of Medical Sciences and Peking Union Medical College, Beijing, China

**Keywords:** blood-retinal barrier, immunometabolic regulation, mitochondrial stress, systemic-ocular crosstalk, TSPO

## Abstract

The eye is increasingly recognized as a tissue shaped by systemic immune and metabolic states rather than an isolated immune-privileged organ. Translocator protein (TSPO), an 18-kDa outer mitochondrial membrane protein enriched in myeloid and glial cells, links mitochondrial homeostasis, redox signaling, lipid metabolism, and immune polarization. This review synthesizes evidence for TSPO involvement in systemic-ocular immunometabolic crosstalk and organizes it into a three-stage working model: peripheral immunometabolic priming, blood-retinal barrier vulnerability, and intraocular glial amplification. We review evidence from systemic autoimmune diseases with ocular manifestations, metabolic diseases with ocular complications, and primary ocular diseases with systemic contributions. We then assess the potential of TSPO as an imaging or fluid biomarker and as a therapeutic target. Current evidence suggests that TSPO is a promising immunometabolic node and underscores the need for cross-organ disease models, cell-specific TSPO manipulation, spatial multi-omics, and local ocular delivery strategies.

## 1 Introduction

The traditional view of ocular immunology holds that the eye is a prototypical immune-privileged organ, protected by the blood-retinal barrier (BRB) and the expression of immunosuppressive molecules. Recent research has gradually challenged this isolated perspective. Systemic autoimmune diseases can affect the uvea, retinal vasculature, and optic nerve (1). Diabetes, hyperlipidemia, obesity, and chronic low-grade inflammation can remodel the retinal neurovascular unit (2). Ageing and systemic dysregulation of the complement system also significantly influence the onset and progression of age-related macular degeneration (AMD) (3). Together, these observations indicate that the ocular microenvironment engages in complex and active dialogue with systemic immune and metabolic states. Immunometabolic regulation is therefore emerging as a useful framework for understanding eye disease.

Within this complex regulatory network, the 18-kDa translocator protein (TSPO) exhibits unique integrative properties. TSPO, initially identified as the peripheral benzodiazepine receptor (PBR), localizes primarily to the outer mitochondrial membrane. Under physiological conditions, it participates in cholesterol transport across the mitochondrial membrane and in steroid hormone synthesis (4–6). Under pathological conditions, TSPO expression is markedly upregulated in activated microglia and macrophages, which has made it a PET imaging biomarker of neuroinflammation (7). However, TSPO functions extend well beyond that role. Accumulating evidence links TSPO to mitochondrial reactive oxygen species (ROS) regulation (8), modulation of NLRP3 inflammasome assembly (9), and shifts in myeloid inflammatory states (10, 11). Taken together, these findings support a cautious view of TSPO as an immunometabolic integrator with potential relevance to systemic-ocular crosstalk (12, 13).

However, the role of TSPO in systemic-ocular immunometabolic crosstalk has not been systematically synthesized. This review proposes a TSPO-centered working framework. We first summarize the molecular and cellular basis of TSPO biology. We then integrate these mechanisms into a three-stage model comprising peripheral immunometabolic priming, BRB vulnerability, and retinal glial amplification, each representing a potential site of TSPO-related mitochondrial, redox, lipid, and immune regulation. We next critically examine TSPO-related evidence across three disease scenarios: systemic autoimmune diseases with ocular manifestations, metabolic diseases with ocular complications, and primary ocular diseases with systemic contributions. Finally, we assess the clinical translation prospects of TSPO as a biomarker and therapeutic target, and identify key gaps in the current evidence base and priority research directions.

This narrative review was informed by structured searches of PubMed and Embase from database inception through August 2026, covering complementary ocular, systemic immunometabolic, mechanistic, and ligand-specific TSPO evidence domains. Given the heterogeneity of study designs, evidence was profiled descriptively according to biological directness to the ocular question (D1–D5) and strength of TSPO attribution (T0–T3). Full search strategies and scope definitions are provided in the **Supplementary Methods**.

## 2 TSPO as an immunometabolic integrator

### 2.1 Molecular structure and functional positioning

TSPO is a highly conserved 18-kDa outer mitochondrial membrane protein with five transmembrane helices and a cholesterol-binding pocket (4, 5, 14). Although it was first framed as a gatekeeper of steroidogenesis, genetic studies showing that global Tspo deletion does not abolish baseline steroid hormone production have shifted the field away from an obligate steroidogenic model. The emerging consensus has repositioned TSPO as a context-dependent regulator of mitochondrial stress, lipid trafficking, and inflammatory signaling (6, 15, 16). These functions are potentially relevant to the eye, where the photoreceptors, glia, epithelial barriers, and vascular cells all depend on mitochondrial integrity and lipid homeostasis.

### 2.2 Tissue and cellular distribution of TSPO

#### 2.2.1 Systemic distribution: from metabolic hubs to peripheral sentinels

TSPO is ubiquitously expressed but highly enriched in tissues with robust lipid metabolism or steroidogenic activity, such as the adrenal glands, heart, and lungs (17). In peripheral blood, TSPO is most readily detected in monocyte-rich fractions, where its expression levels appear to vary with the systemic inflammatory tone (13). This peripheral enrichment positions TSPO as a vital immunometabolic signal that can be modulated during systemic stress before any overt tissue damage occurs.

#### 2.2.2 Ocular distribution: constitutive versus inducible profiles

Within the eye, TSPO is expressed predominantly in glial and barrier-associated cells rather than neurons. Across human and mouse retina, TSPO has been detected in Müller glia, microglia, vascular cells, and retinal pigment epithelium (RPE), with comparatively low expression in retinal neurons (7, 12, 18–21).

TSPO distribution follows a distinct functional duality across different cell compartments. In healthy tissue, constitutive expression is most evident in RPE and vascular compartments, consistent with their roles in lipid transport, redox buffering, and barrier maintenance (12, 19, 20). By contrast, in injury or degeneration, TSPO becomes more prominent in reactive microglia and Müller cells, linking its inducibility to gliosis and local innate immune activation (7, 21, 22). This pattern suggests a functional split: constitutive TSPO supports barrier homeostasis, whereas inducible TSPO marks reactive glial remodeling.

### 2.3 Key TSPO-mediated signaling pathways

Across ocular tissues and systemic immune cells, TSPO biology can be organized around four interconnected mechanisms: mitochondrial homeostasis, redox and inflammasome signaling, metabolic polarization, and cholesterol handling. Within this framework, altered Ca^2+^ handling reshapes mitochondrial and NOX-dependent ROS production, redox stress can promote inflammasome activation and lipid peroxidation, and impaired cholesterol efflux can feed back on mitochondrial fitness and immune-cell state. Representative mechanistic evidence supporting these interconnected TSPO pathways is summarized in **Table 1**.

**Table 1:** Representative mechanistic evidence linking TSPO to ocular and systemic immunometabolism context.

Mechanistic domain	Model/system	TSPO modulation or readout	Key finding	Evidence Profile^a^	Study
RPE lipid metabolism	Human RPE cells; aged mouse RPE/choroid	TSPO ligands; TSPO knockdown/loss	TSPO ligands promote cholesterol efflux, whereas TSPO loss increases oxLDL uptake, lipid accumulation, ROS and inflammatory cytokines.	D2/4; T2	Biswas et al., 2017 (12)
Choroidal endothelial inflammation	Primate choroidal endothelial cells	Etifoxine; XBD173	TSPO ligands increase cholesterol efflux and reduce oxLDL-induced ROS, VEGF and inflammatory cytokine release.	D4; T1	Biswas et al., 2018 (19)
RPE metabolic remodeling	ARPE-19 cells	TSPO knockout	TSPO knockout cells alter fatty-acid and glycerophospholipid metabolism and increase GSSG	D4; T2	Alamri et al., 2019 (34)
RPE antioxidant response	ARPE-19 cells	XBD173, PK11195, Ro5-4864	TSPO ligands increase Nrf2/HMOX1-associated defense and reduce ROS/inflammasome activity	D4; T1	Rashid et al., 2020 (35)
RPE oxidative injury	ARPE-19 cells	TSPO genetic deletion	TSPO deletion reduces TRPM2/PARP-1-associated Ca²^+^ signaling and oxidative cell death.	D4; T2	Özkaya et al., 2021 (20)
Diabetic endothelial stress	HFD/STZ diabetic mouse retina; high-glucose hRMECs	Ginkgolide B; TSPO/Nrf2 pathway	Ginkgolide B suppresses endothelial ferroptosis and improves DR-related vascular/barrier changes through TSPO/Nrf2 signaling.	D2/3; T1	Lu et al., 2026 (36)
Reactive retinal microglia	Human and mouse retinal microglia	TSPO expression/function	TSPO is expressed in reactive retinal microglia and modulates microglial inflammatory response, migration and phagocytosis.	D1/2/4; T2	Karlstetter et al., 2014 (7)
Retinal degeneration	Light-induced retinal degeneration	XBD173; PK11195	TSPO ligands dampen pro-inflammatory microglial reactivity and protect photoreceptors.	D2; T1	Scholz et al., 2015 (21)
Retinal ischemia	Mouse retinal ischemia	XBD173	XBD173 attenuates microglial/Müller glial response and protects inner retinal neurons.	D2; T1	Mages et al., 2019 (22)
Pathological angiogenesis	Laser-CNV and ocular phagocyte models	TSPO genetic deletion/modulation; NOX1 axis	The TSPO–NOX1 axis controls phagocyte-triggered ROS generation and pathological angiogenesis.	D2/3; T1	Wolf et al., 2020 (27)
Photoreceptor-like inflammatory injury	661W photoreceptor-like cells	PIGA ligands	PIGA ligands reduce LPS-induced inflammatory and apoptotic responses in photoreceptor-like cells.	D4; T1	Corsi et al., 2022 (37)
Retinitis pigmentosa model	rd10 mouse model	Topical PIGA1138	Topical PIGA1138 preserves retinal structure/function and reduces inflammatory/apoptotic readouts.	D2; T1	Corsi et al., 2025 (38)
Peripheral TSPO in MS	PBMCs from MS patients	Peripheral TSPO expression/binding	Monocytes account for most PBMC TSPO signal; peripheral TSPO is lower in MS patients than controls.	D5; T1	Harberts et al., 2013 (13)
Human macrophage polarization	Human monocyte-derived macrophages	M1/M2 polarization; TSPO expression	Pro-inflammatory M1 stimulation downregulates TSPO mRNA and protein; M2 effects are source- and condition-dependent.	D5; T1	Narayan et al., 2017 (32)
Microglia bioenergetics	Human C20 microglial cells	TSPO knockout	TSPO knockout alters mitochondrial respiration and cellular metabolism	D5; T2	Milenkovi et al., 2019 (26)
Microglia activation	Primary mouse microglia BV-2	Germline Tspo knockout; shRNA	TSPO deficiency impairs oxidative phosphorylation and attenuates activation	D5; T2	Yao et al., 2020 (33)
Microglia/macrophage M2 regulation	Primary microglia/macrophages	IL-4; PK11195; FGIN-1-27; TSPO overexpression	IL-4-induced M2 polarization is associated with decreased TSPO protein; PK11195 promotes M2 markers, whereas FGIN-1–27 and TSPO overexpression suppress M2 markers.	D5; T2	Zhou et al., 2020 (10)
Systemic neuroinflammation monitoring	Irradiated mice	TSPO-high monocytes	Peripheral TSPO-high monocytes correlate with brain TSPO-high cells after irradiation.	D5; T1	Rodina et al., 2023 (39)
Plasma TSPO in sepsis	Human sepsis patients	Plasma TSPO	Plasma TSPO differs between sepsis patients and controls; its severity association appears limited.	D5; T1	Hattori et al., 2024 (40)

a Evidence profile: D1–D5 indicate progressively decreasing biological directness to the ocular question, whereas T0–T3 indicate progressively increasing strength of TSPO attribution. These descriptors are intended to facilitate interpretation of heterogeneous evidence and do not represent an overall methodological-quality or certainty score. Full definitions are provided in the **Supplementary Methods**.

TSPO, translocator protein; RPE, retinal pigment epithelium; BRB, blood–retinal barrier; oxLDL, oxidized low-density lipoprotein; ROS, reactive oxygen species; VEGF, vascular endothelial growth factor; TRPM2, transient receptor potential melastatin 2; PARP-1, poly(ADP-ribose) polymerase 1; HFD, high-fat diet; STZ, streptozotocin; hRMECs, human retinal microvascular endothelial cells; Nrf2, nuclear factor erythroid 2–related factor 2; DR, diabetic retinopathy; PBMCs, peripheral blood mononuclear cells; MS, multiple sclerosis; CNV, choroidal neovascularization; NOX1, NADPH oxidase 1; LPS, lipopolysaccharide; IL-4, interleukin-4; M1/M2, classically/alternatively activated macrophages; CNS, central nervous system.

#### 2.3.1 The Ca^2+^-ROS axis and mitochondrial homeostasis

TSPO is closely linked to mitochondrial bioenergetics and organelle quality control. Located in the mitochondrial outer membrane, TSPO acts as a pivotal regulator of Ca^2+^ flux and mitochondrial physiology. Recent knockout studies demonstrate that TSPO deficiency impairs basal and maximal respiration, reduces mitochondrial membrane potential, and compromises calcium buffering capacity, leading to increased mitochondrial fragmentation. Conversely, TSPO overexpression or targeted modulation can reshape mitochondrial bioenergetics, influencing the threshold at which these organelles transition from homeostatic support to stress-induced dysfunction (23–25). Changes in mitochondrial Ca^2+^ buffering therefore provide a proximal link between TSPO-associated bioenergetic control and downstream redox signaling (26).

Under retinal injury, this Ca2+-sensitive regulatory network extends beyond mitochondrial homeostasis to NADPH oxidase 1(NOX1)-dependent ROS production in retinal phagocyte. Specifically, TSPO appears to gate the Ca^2+^-dependent activation of NOX1. Genetic deletion of TSPO reduces NOX1-driven ROS production and shifts these cells away from a neurotoxic phenotype (27). By increasing the intracellular ROS burden, this pathway may also lower the threshold for redox-sensitive inflammasome and cytokine signaling (28).

#### 2.3.2 NLRP3 inflammasome and cytokine signaling

Mitochondrial and NOX-derived ROS can provide upstream signals that facilitate inflammatory activation. TSPO thus represents a critical molecular rheostat that shapes the threshold at which mitochondrial stress is translated into cytokine production and tissue damage. In ARPE-19 cells, TSPO deficiency has been shown to reduce mitochondrial ROS production, TRPM2 activation, and subsequent oxidative cell death, linking TSPO to redox-sensitive injury signaling in the outer retina (20).

This regulatory role extends to inflammatory amplification pathways. Direct TSPO-dependent regulation of NLRP3 is best supported in neuronal OGD/R models, where TSPO knockdown reduces inflammasome activity and apoptosis (29). In retinal injury models such as light damage and ischemia, TSPO ligands alleviated neuron cell damage by reducing pro-inflammatory factors (21, 22). Together, these findings demonstrate that precise TSPO modulation can dampen downstream inflammatory escalation. At a broader immunometabolic level, inflammasome activity and cholesterol handling form a reciprocal loop. Defective ABCA1/ABCG1-mediated efflux can promote cholesterol accumulation and NLRP3 activation, whereas IL-1beta-driven inflammation can further impair ABCA1-dependent efflux (30, 31). This feedback provides the mechanistic bridge to the lipid axis discussed below.

#### 2.3.3 The ABCA1-lipid axis and cholesterol homeostasis

At the ocular barrier interface, this redox-inflammatory feedback intersects with TSPO-associated cholesterol transport. Within the RPE and choroidal endothelial cells, TSPO acts as a mitochondrial lipid-stress buffer. In RPE cells, TSPO ligands increase cholesterol efflux to lipoprotein acceptors, while TSPO loss promotes oxidized LDL uptake, intracellular lipid accumulation, ROS generation, and inflammatory cytokine expression (12). Similar effects have been demonstrated in choroidal endothelial cells, where TSPO ligands increase ABCA1/ABCG1-associated cholesterol export, reduce VEGF and inflammatory cytokines, and improve antioxidant capacity (19). These findings place TSPO at the intersection of sub-RPE lipid deposition, oxysterol stress, and endothelial dysfunction.

#### 2.3.4 Immunometabolic reprogramming and phenotypic polarization

Beyond its homeostatic role, TSPO has also been linked to context-dependent metabolic reprogramming in immune cells. Specifically, TSPO expression decreased during IL-4-induced restorative activation in primary microglia (10). Meanwhile, it can also be downregulated during classical pro-inflammatory activation in human macrophage, whereas the tested M2 stimuli did not significantly alter its expression (32). Notably, genetic TSPO deficiency attenuated both IL-4- and LPS-induced microglial activation by impairing mitochondrial membrane potential, ATP production, oxidative phosphorylation, and glycolysis, indicating that reduced TSPO expression during polarization should not be equated with complete TSPO loss (33). In the outer retina, such metabolic plasticity enables glial and barrier-associated cells to adapt to lipid stress while maintaining tissue homeostasis.

## 3 Mechanisms of TSPO-mediated systemic-ocular immunometabolic crosstalk

Direct evidence that TSPO alone drives a complete systemic-to-ocular disease axis remains limited. A more defensible conceptualization frames current data within a three-step working model: peripheral immunometabolic priming, barrier vulnerability, and intraocular amplification. The evidential strength varies across these stages. Human data are relatively stronger for peripheral TSPO alterations and diabetic retinopathy-associated biomarker signals, while direct ocular mechanistic evidence comes mainly from retinal degeneration, ischemia, diabetes, and barrier-cell experiments. Collectively, we proposed a TSPO-centered conceptual framework in which systemic inflammatory stress primes circulating immune signals, constitutive TSPO deficiency weakens the metabolic resilience of ocular barrier cells, and inducible TSPO upregulation in retinal glia influences whether the intraocular response achieves resolution or drives self-amplifying pathology. The proposed three-stage TSPO-centered framework is illustrated in **Figure 1**.

**Figure 1:**
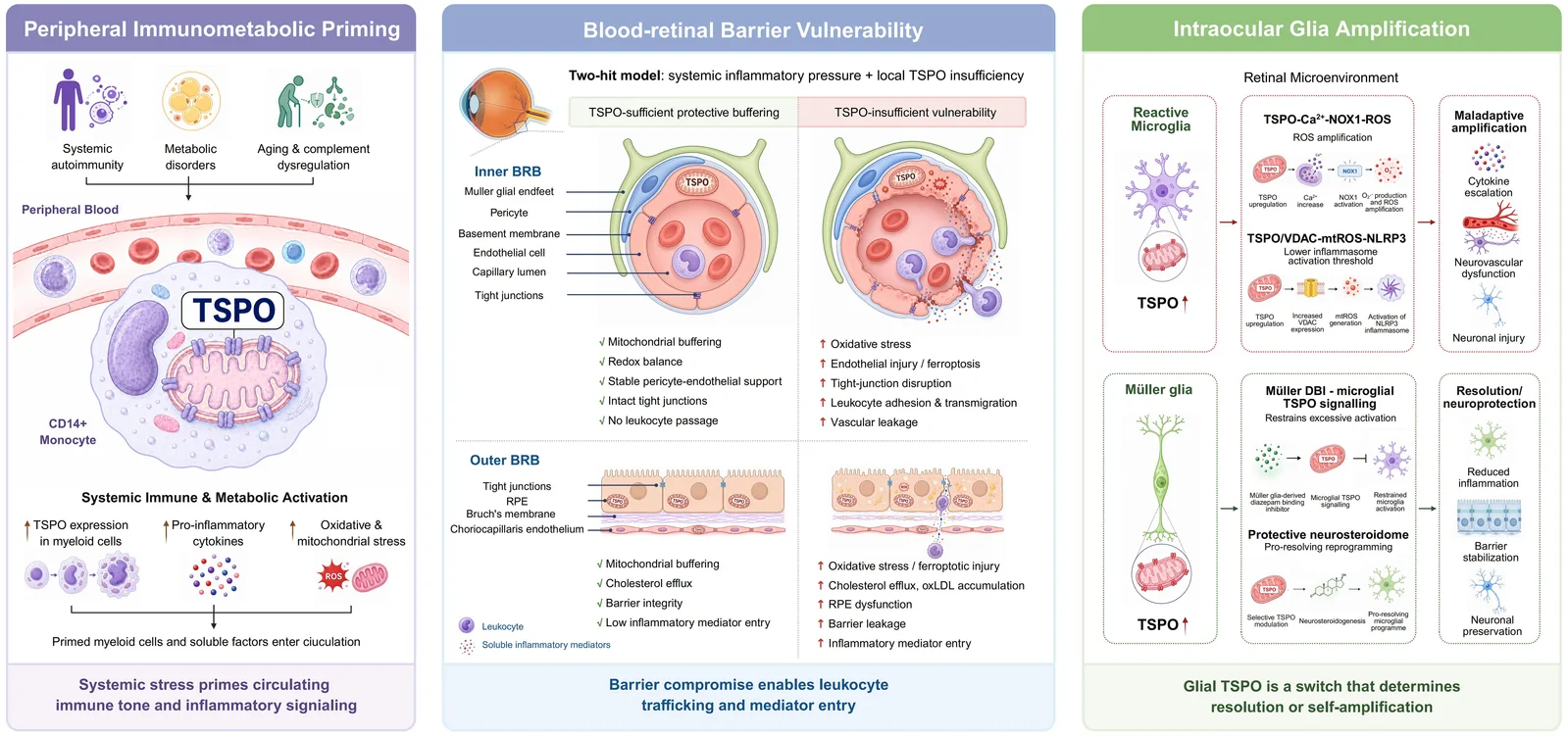
Conceptual framework of TSPO-mediated systemic-ocular immunometabolic crosstalk. The three-stage model traces how systemic stress propagates to ocular pathology through TSPO-dependent mechanisms. Stage 1 (peripheral immunometabolic priming): circulating monocytes and macrophages exhibit state-dependent TSPO changes that report systemic inflammatory and metabolic burden. Stage 2 (BRB vulnerability transition): constitutive TSPO in retinal endothelial cells, RPE, and choroidal endothelial cells maintains barrier resilience through cholesterol efflux, redox buffering, and ferroptosis suppression; loss of this capacity allows systemic stress to breach the barrier. Stage 3 (intraocular glial amplification): inducible TSPO upregulation in reactive microglia and Müller glia determines whether the local response resolves or self-amplifies into chronic neuroinflammation.

### 3.1 Peripheral immunometabolic priming

#### 3.1.1 TSPO as a peripheral immunometabolic signal

The first stage of the model is systemic priming. In human peripheral blood mononuclear cells, CD14+ monocytes account for most measurable TSPO (13), which indicates that the protein is a myeloid-enriched state indicator.

Peripheral TSPO is highly dynamic and varies with the systemic inflammatory context. In multiple sclerosis (MS), peripheral TSPO measured by radioligand binding is significantly lower than in healthy controls (13). In sepsis, a recent case-control study also reported significantly lower plasma TSPO concentrations in patients (40). By contrast, acute inflammatory stimulation can increase TSPO display on monocytes. For instance, *in vitro* lipopolysaccharide (LPS) exposure increased the fraction of circulating monocytes with surface TSPO expression (11). Taken together, these findings indicate that peripheral TSPO can reflect the metabolic and activation state of circulating myeloid cells, and the systemic inflammatory burden in the human body.

#### 3.1.2 Context-dependent TSPO regulation in monocyte polarization

Mechanistically, peripheral TSPO appears to shape immune-cell state rather than simply label activated cells. TSPO knockout in cultured microglia disrupts mitochondrial respiration and calcium buffering (26). At the gene level, TSPO expression behaves in a context-dependent manner: in human macrophages, a classical pro-inflammatory polarization downregulated TSPO, whereas an alternative (IL-4-driven) polarization did not significantly alter TSPO (10, 32).

Metabolic diseases provide a clinically relevant extension for this priming concept. In patients with diabetic retinopathy (DR), PBMCs exhibit a pro-inflammatory signature characterized by upregulation of TSPO, VDAC, and key inflammasome components (NLRP3, ASC, caspase-1). These signals intensify in active proliferative DR and correlate with clinical disease severity (9). In addition, serum TSPO has also been associated with hyperreflective retinal spots (HRS) on OCT, a potential marker of retinal microglial activation and early neurovascular injury (41).

Experimental imaging adds tissue-level support. In a recent [18F]-DPA-714 study, primary microglia/macrophages isolated from neonatal rat cornea, retina, choroid, and sclera showed increased tracer uptake after high-glucose exposure, and streptozotocin-diabetic rats showed ocular TSPO-related PET signal as early as 3 to 6 weeks. These findings suggest that diabetic stress can prime ocular myeloid TSPO responses before advanced structural disease develops (42).

Taken together, these observations support a peripheral immunometabolic role for TSPO: systemic immune tone and mitochondrial stress are reflected in TSPO-linked signals that may precede endothelial, leukocyte, and glial changes in the retina.

### 3.2 Blood-retinal barrier vulnerability

The second stage of this crosstalk framework is barrier vulnerability. TSPO serves as a constitutive metabolic buffer within the barrier structural cells. When this capacity is diminished, systemic inflammatory pressure more easily translates into overt blood-retinal barrier (BRB) dysfunction.

#### 3.2.1 The inner BRB: retinal endothelial integrity and ferroptosis control

The inner BRB, formed by the tight junctions of retinal microvascular endothelial cells, is a plausible downstream site of TSPO-related metabolic vulnerability. Ferroptosis, an iron-dependent form of regulated cell death, is driven by unrestrained phospholipid peroxidation when endogenous antioxidant defenses fail to detoxify lipid hydroperoxides. Mechanistically, acyl-CoA synthetase long-chain family member 4 (ACSL4) enriches cellular membranes with peroxidation-prone polyunsaturated phospholipids, whereas glutathione peroxidase 4 (GPX4) counteracts ferroptotic injury by reducing lipid hydroperoxides. Glucolipotoxic stress can therefore sensitize retinal endothelial cells to ferroptosis by increasing oxidative pressure while weakening Nrf2-associated antioxidant defense (43).

Recent evidence has examined TSPO in this context. In high-fat diet/streptozotocin-induced diabetic mice and HG/PA-challenged hRMECs, Lu et al. reported that glucolipotoxicity suppresses TSPO/Nrf2 signaling, thereby accelerating ferroptotic endothelial injury. Ginkgolide B restored TSPO/Nrf2 pathway, reduced ferroptosis-associated changes, and improved vascular barrier function. Conversely, pharmacological inhibition of TSPO with PK11195 blunted Ginkgolide B-mediated protection, downregulating Nrf2 and GPX4 while upregulating ACSL4 (36). These findings support a model in which endothelial TSPO helps preserve the inner BRB by linking mitochondrial stress handling to lipid-peroxide detoxification. However, direct ginkgolide B-TSPO binding and molecular coupling between TSPO and Nrf2 remain unproven.

#### 3.2.2 The outer BRB: The RPE-choroid metabolic interface

The outer BRB, consisting of the RPE and the underlying choroidal endothelium, provides stronger mechanistic evidence for TSPO involvement. In human RPE cells, TSPO ligands enhance cholesterol efflux to apolipoproteins, whereas TSPO loss increases oxidized LDL uptake, intracellular lipid accumulation, ROS production, and inflammatory cytokines release. Moreover, TSPO-dependent oxidative cytotoxicity in ARPE-19 cells appears to involve modulation of TRPM2, linking TSPO to a TRPM2-Ca2+-ROS injury axis (20, 34). Importantly, aged mouse RPE cells show reduced TSPO expression together with impaired cholesterol efflux (12). Consistent with this interpretation, Tspo knockout mice show increased cholesterol, triglycerides, and phospholipids in RPE cells, downregulation of cholesterol homeostasis genes such as Lxra, Abca1, Abcg1, Cyp27a1, and Cyp46a1, and increased retinal inflammatory cytokines (44).

Parallel evidence has been reported in choroidal endothelial cells. TSPO ligands such as etifoxine and XBD173 increase cholesterol efflux, upregulate LXRa/CYP27A1/CYP46A1/ABCA1/ABCG1, reduce cholesterol and phospholipid biosynthesis, lower oxLDL-induced ROS, and suppress IL-1beta, IL-6, TNF-alpha, and VEGF release (19). Collectively, these studies support the view that TSPO may contribute to barrier metabolic resilience at the RPE-choroid interface.

#### 3.2.3 The two-hit model of barrier destabilization

A reasonable synthesis is a two-hit model of BRB failure. The first hit is systemic: inflammation, hyperglycemia, or lipotoxic stress primes circulating immune cells and increases the pressure placed on the ocular barrier. The second hit is local: insufficient TSPO-mediated mitochondrial and lipid buffering in retinal endothelial cells, RPE cells, and choroidal endothelial cells reduces the capacity of the barrier to absorb that pressure. In this framework, TSPO does not represent the entire barrier lesion by itself; rather, it determines how readily systemic stress is translated into barrier breakdown.

### 3.3 Intraocular amplification loop

#### 3.3.1 Glial reactivity as a switch point for inflammation

Once the BRB becomes permissive, a local amplification loop is initiated. Infiltrating or systemically primed signals then act as a second hit on a retina already predisposed to reactivity. Within this phase, inducible TSPO upregulation in retinal glia may influence whether inflammation remains constrained or escalates into self-propagating neuroinflammation.

Studies show that TSPO is present in human retinal microglia and is strongly upregulated in reactive retinal microglia during degeneration, making it a plausible marker of glial reactivity in diseased retina (7). Experimental intervention further suggests that TSPO is not merely a bystander, as pharmacological targeting with TSPO ligands directly tunes these cellular responses. Specifically, Scholz et al. showed that XBD173 dampened pro-inflammatory microglial activation, reduced cytokine release, and protected photoreceptors in a light-damage model. Similarly, Mages et al. demonstrated that the ligand XBD173 attenuated microgliosis and preserved inner retinal neurons after ischemia (21, 22). More recently, topical TSPO modulation with PIGA1138 preserved retinal function and structure in rd10 mice, delayed photoreceptor loss, and reduced inflammatory and apoptotic readouts, extending the same principle to inherited retinal degeneration (38). These findings support that TSPO can influence the balance of glial response. A comparative synthesis of these pharmacologically heterogeneous TSPO ligands/modulators is provided in Section 4.2.1.

#### 3.3.2 Müller glia-microglia immunometabolic restraint

The amplification phase also involves Müller glia-microglia interactions. In the retina, TSPO is present in Müller glia, where it may contribute to local restraint of microglial activation. In mouse models, the retinal DBI-TSPO axis from Müller glia has been reported to suppress microglial inflammatory output, providing a plausible mechanistic bridge between TSPO signaling and local restraint of innate immunity (8). This DBI-TSPO axis has also been examined in neovascular retinopathy. Gao et al. combined vitreous samples from patients with proliferative diabetic retinopathy, an oxygen-induced retinopathy model, and Müller glia-microglia experiments. Anti-VEGF treatment was associated with induced DBI-TSPO signaling, reduced microglial activation, and a more neurotrophic and anti-inflammatory response (45). The multimodal design strengthens the biological plausibility of Müller glia-microglia restraint.

Mechanistically, this regulation does not simply silence the immune response but may promote a functional reprogramming of retinal phagocytes. In LPS-challenged BV-2 microglia, TSPO modulation can suppress inflammatory toxicity (46). Beyond acute anti-inflammatory effects, TSPO engages a restorative program by supporting *de novo* neurosteroidogenesis and BDNF-related trophic signaling (47). Recent evidence suggests that selective TSPO modulation can shift human microglia toward a protective neurosteroidome, characterized by anti-inflammatory and reparative steroid profiles (48). These findings support the idea that TSPO may help uncouple inflammatory cytotoxicity from essential homeostatic functions, thereby influencing whether the intraocular response remains self-limited or becomes persistent.

## 4 Cross-disease evidence

### 4.1 Systemic immune diseases with ocular involvement

Systemic immune diseases provide an important validation scenario for TSPO-related systemic-ocular crosstalk. The following two clinical settings illustrate different facets of this crosstalk. Multiple sclerosis (MS) demonstrates the directional dissociation between peripheral and central TSPO signals and its association with optic neuritis. Vasculitis and uveitis offer a clinical setting in which vascular wall inflammation can propagate to intraocular tissue, but TSPO-specific evidence in these conditions remains preliminary. The TSPO evidence across the major systemic-ocular disease categories discussed below is summarized in **Table 2**.

**Table 2:** Summary of TSPO evidence across systemic-ocular disease categories.

Disease	Evidence type	System/cell studied	Key finding	Study & evidence profile
MS	TSPO-PET; PBMC TSPO	CNS lesions; peripheral monocytes	Peripheral TSPO reduced, CNS TSPO elevated; chronic active lesion signal	Polvinen et al., 2023 (51), D5, T1; Nutma et al., 2019 (53), D5, T1; Harberts et al., 2013 (13), D5, T1
Vasculitis/Uveitis	TSPO-PET	CNS vasculature	Regional signal in primary CNS vasculitis; ocular TSPO data limited	Backhaus et al., 2020 (56), D5, T1
DR	PBMC TSPO; serum TSPO; PET; cell models	PBMCs; serum; rat retina; endothelial cells	TSPO-VDAC-NLRP3 up in PDR; serum TSPO links to HRS; early ocular PET signal; TSPO/Nrf2 ferroptosis control	Guo et al., 2022 (9), D5, T1; Trotta et al., 2022 (41), D1/5, T1; Chen et al., 2024 (42), D2, T1; Lu et al., 2026, D2/3, T1 (36)
AMD	Cell models; mouse KO	RPE; choroidal ECs; mouse retina	TSPO ligands promote cholesterol efflux; TSPO loss causes lipid accumulation; TSPO-NOX1 in angiogenesis	Biswas et al., 2017 (12), D2/4, T2; Biswas et al., 2018 (19), D4, T1; Farhan et al., 2021 (44), D2/3, T2; Wolf et al., 2020 (27), D2/3, T1
Glaucoma	Ex vivo model; ischemia model	Rat retina; mouse retina	Pressure-induced TSPO upregulation; TSPO ligand promotes neurosteroid protection; XBD173 reduces gliosis	Ishikawa et al., 2016 (64), D3, T1; Mages et al., 2019 (22), D2, T1; Zeng et al., 2023 (67), D2/4/5, T2

AE, autoimmune encephalitis; AMD, age-related macular degeneration; CNS, central nervous system; DR, diabetic retinopathy; EC, endothelial cell; HRS, hyperreflective spots; KO, knockout; MS, multiple sclerosis; PBMC, peripheral blood mononuclear cell; PET, positron emission tomography; RPE, retinal pigment epithelium.

#### 4.1.1 Multiple sclerosis and optic neuritis

MS is a representative autoimmune demyelinating disease of the central nervous system (CNS) and an important clinical model for studying TSPO-related neuroinflammation imaging and visual pathway damage. Optic neuritis is one of the most common visual system manifestations of MS: approximately 20% of MS patients present with optic neuritis as the initial symptom, and approximately 50% develop optic neuritis during the disease course (49). Beyond acute optic neuritis, OCT studies have shown that MS patients, even without a documented history of optic neuritis, can exhibit thinning of the retinal nerve fiber layer (RNFL) and the macular ganglion cell-inner plexiform layer (GCIPL) (13, 50). This suggests that MS-related retinal changes reflect not only secondary injury following focal optic nerve demyelination, but also the extension of CNS chronic inflammation and neurodegeneration into the visual system.

The significance of TSPO in MS can be considered in two respects. First, CNS TSPO-PET can reveal chronic inflammatory activity that conventional MRI cannot fully capture. [11C]PK11195 and other TSPO tracers have shown that persistent TSPO signal elevation can occur at the edges of chronic active lesions and in normal-appearing white matter, consistent with chronic innate immune activation associated with microglia and macrophages (51, 52). Nutma et al. established a direct correspondence between TSPO-PET signals and microglia/macrophage density in post-mortem tissue through quantitative neuropathological assessment (53). Second, peripheral and CNS TSPO signals do not necessarily change in parallel. Harberts et al. found that TSPO expression in PBMCs of MS patients was lower than in healthy controls, while CNS TSPO-PET signal could be elevated (13). This peripheral-central dissociation suggests that TSPO cannot be interpreted as a uniform biomarker across different compartments.

The peripheral-central TSPO dissociation directly supports the three-stage model hypothesis that peripheral immunometabolic priming is not identical to intraocular amplification. Peripheral blood TSPO alone cannot be assumed to predict concordant intraocular change: peripheral downregulation may reflect immunometabolic reprogramming of systemic myeloid cells, while CNS or intraocular resident microglial TSPO upregulation may indicate local neuroinflammatory activation. A more reasonable strategy is to jointly analyze peripheral immune markers, optic nerve and visual pathway MRI, OCT-measured RNFL/GCIPL changes, and, when necessary, TSPO-PET. Such multimodal studies help distinguish three types of processes: systemic immune state changes, local glial activation, and retinal neuroaxonal degeneration.

#### 4.1.2 Systemic immune diseases with uveal and retinal vascular involvement

Whereas MS primarily involves CNS inflammation and visual pathway involvement, systemic immune disease-related uveitis, retinal vasculitis, and ischemic optic neuropathy more directly involve intraocular tissue, ocular vasculature, and the blood-ocular barrier. This spectrum of ocular involvement broadly includes two overlapping clinical scenarios. The first is systemic vasculitis-related eye disease, such as giant cell arteritis, ANCA-associated vasculitis, and Behcet disease, which can affect the optic nerve, retinal vasculature, and uveal tissue through vascular wall inflammation, endothelial damage, vascular leakage, or tissue ischemia. The second is non-vasculitic systemic immune disease-related uveitis, such as sarcoidosis, spondyloarthropathy, and inflammatory bowel disease-associated uveitis, primarily manifested as blood-ocular barrier disruption, intraocular inflammatory cell infiltration, cytokine network activation, retinal vascular leakage, and macular oedema (54, 55). These diseases therefore provide clinically relevant settings for observing the connections between systemic immune status, ocular barrier function, and the local inflammatory microenvironment.

Currently, direct evidence for TSPO in systemic immune-related uveitis and retinal vasculitis is limited. Studies have shown that [18F]DPA-714 TSPO-PET can identify regional signals associated with inflammatory lesions in primary CNS vasculitis, suggesting that TSPO imaging may reflect myeloid cell responses associated with vascular inflammation (56). In parallel, retinal studies show that TSPO is upregulated in reactive microglia and can participate in microglial inflammatory responses, migration, and phagocytic function; the TSPO ligand XBD173 can reduce glial reactivity and protect inner retinal neurons in retinal ischemia models (21, 22). These results suggest a mechanistic association between TSPO and vascular inflammation, retinal glial reactivity, and neurovascular stress, though direct evidence in these conditions remains preliminary.

### 4.2 Diabetic retinopathy: a paradigm of metabolic-ocular crosstalk

Diabetic retinopathy (DR) is a representative disease in which systemic metabolic abnormalities affect ocular structure and function through the BRB and the retinal neurovascular unit. DR has long been regarded as a diabetes-related microvascular complication, but current research has shown that its pathological process is not limited to microvascular damage. It also includes retinal neurodegeneration, Müller cell and microglial reactivity, chronic low-grade inflammation, oxidative stress, and BRB dysfunction (57, 58). DR therefore provides a relatively clear clinical and experimental model for analyzing the continuum between systemic metabolic stress, barrier dysfunction, and intraocular inflammation and glial reactivity.

Compared with systemic immune diseases, TSPO evidence in DR more directly connects peripheral immunometabolic status with intraocular inflammatory responses. Peripheral TSPO evidence in DR converges on three levels. At the PBMC expression level, Guo et al. found that TSPO, VDAC, and NLRP3 inflammasome-related molecules were higher in PBMCs of DR patients than in age-related cataract controls. Within DR, TSPO and VDAC expression were higher in proliferative DR (PDR) than in non-proliferative DR (NPDR), and inflammation markers were also higher in active PDR than in inactive PDR (9). At the serum biomarker level, Trotta et al. detected serum Iba-1, GLUT5, and TSPO in an exploratory clinical study, finding that these microglia-related markers correlated with OCT hyperreflective spots (HRS) and retinal thickness changes (41). Since HRS is often used as an imaging surrogate for retinal microglia/macrophage activation or lipid/inflammatory deposition, this study suggests that serum TSPO may reflect the connection between systemic inflammatory status and retinal neurovascular changes in DR. However, this study had a limited sample size, and HRS is not a TSPO-specific imaging marker; larger and longitudinal studies are needed for validation. At intraocular fluid level, vitreous DBI in PDR was associated with VEGF, inflammatory, and neurotrophic mediators, providing an additional link between intraocular vascular activity and the Müller glia-microglia TSPO axis (45).

Animal and cell studies provide tissue-level evidence for TSPO involvement in diabetic ocular inflammation. Chen et al. reported increased retinal [18F]DPA-714 uptake in the early phase of diabetic rat modelling (3 and 6 weeks), consistent with intraocular microglia/macrophage activation and TSPO upregulation (42); whereas Zhou et al. detected reduced ocular tracer at 12 weeks after STZ-induced diabetes, suggesting that TSPO level may play an important role in the development of DR (59). In addition, recent research has shown that ginkgolide B can inhibit retinal vascular endothelial cell ferroptosis through the TSPO/Nrf2 pathway and improve vascular endothelial damage and BRB-related changes in DR models (36). These results suggest that TSPO may participate in intraocular myeloid/glial cell responses and endothelial barrier stress under diabetic conditions.

Overall, DR provides relatively coherent TSPO evidence for the systemic-ocular immunometabolic crosstalk framework proposed in this review: PBMC studies suggest peripheral immunometabolic priming, serum biomarker studies connect circulating TSPO with OCT neuroinflammatory phenotypes, animal PET studies show early ocular TSPO-related inflammatory signals in diabetes, and endothelial cell studies suggest that TSPO may participate in BRB protection and mitochondrial stress regulation. However, this evidence chain still requires paired longitudinal data from peripheral blood, aqueous humor/vitreous, retinal imaging, and treatment response in the same patients for further validation.

### 4.3 Primary ocular diseases with systemic components

#### 4.3.1 Age-related macular degeneration

Age-related macular degeneration (AMD) is an important cause of central vision impairment in older adults. Its main pathological features include drusen formation, RPE dysfunction, Bruch membrane lipid deposition, choroidal capillary abnormalities, and photoreceptor degeneration (60). AMD primarily manifests as degenerative disease of the macular region, but its onset and progression are significantly modulated by systemic genetic, metabolic, and inflammatory backgrounds. Large-scale GWAS studies have shown that AMD risk loci are highly enriched in pathways related to complement regulation, extracellular matrix remodeling, and lipid metabolism, including genes such as CFH, ARMS2/HTRA1, C3, CFI, APOE, and LIPC (3, 61). AMD is therefore not a purely local retinal degeneration, but the result of interaction between primary eye disease and systemic complement-lipid-inflammation susceptibility.

In this context, the potential significance of TSPO in AMD lies primarily in immunometabolic regulation at the outer blood-retinal barrier and the retinal-choroidal interface. The RPE and Bruch membrane region are chronically subjected to lipid deposition, oxidative stress, and complement activation pressure. TSPO, as an outer mitochondrial membrane protein, is associated with cholesterol transport, mitochondrial stress, and inflammation regulation. Studies have shown that TSPO ligands can promote ABCA1/ABCG1-associated cholesterol efflux in RPE cells, while TSPO loss leads to increased oxidized LDL uptake, intracellular lipid accumulation, and pro-inflammatory cytokine release (12). In choroidal endothelial cells, TSPO ligands can also promote cholesterol efflux and reduce oxidized LDL-induced ROS generation and release of inflammatory and vasoactive factors such as IL-6, TNF-alpha, and VEGF (19). These findings suggest that TSPO may participate in lipid handling, oxidative stress regulation, and inflammation modulation in the RPE-Bruch membrane-choroid complex.

TSPO may also be related to retinal myeloid cell responses in AMD. Studies have shown that TSPO is upregulated in reactive retinal microglia and can regulate microglial inflammatory responses, migration, and phagocytic function (7). In retinal degeneration models such as light damage, the TSPO ligand XBD173 can reduce microglial pro-inflammatory responses and glial reactivity, with protective effects on photoreceptors (21). In ocular phagocytes, TSPO-dependent increases in cytosolic Ca²^+^ promote NOX1 expression and activation, leading to extracellular ROS production. This TSPO–NOX1–ROS pathway contributes to phagocyte reactivity, vascular leakage, and choroidal neovascularization. Notably, NOX1 deficiency reduces choroidal neovascularization without altering VEGF-A secretion, indicating that this proangiogenic effect is not mediated by direct stimulation of VEGF production but instead reflects a redox-dependent mechanism that is at least partly independent of changes in VEGF abundance (27). Together, these studies support the possibility of TSPO as a retinal-choroidal immunometabolic stress node.

However, TSPO staging and cellular sources in human AMD eye tissue remain unclear. Early drusen, pre-atrophic, geographic atrophy, and wet AMD may correspond to distinct TSPO states: early stages dominated by RPE lipid handling and antioxidant function, atrophic stages dominated by microglia-macrophage migration and complement activation, and neovascular stages potentially involving a VEGF-inflammation positive feedback loop. Staging-cell co-localization analysis and synergistic evaluation of TSPO modulation with complement inhibition or anti-VEGF therapy are priority directions for future research.

#### 4.3.2 Glaucoma

Glaucoma is a group of chronic neurodegenerative eye diseases characterized by progressive retinal ganglion cell (RGC) loss and optic nerve damage, and is one of the leading causes of irreversible blindness worldwide. Elevated intraocular pressure is currently the most important and modifiable risk factor, but normal-tension glaucoma and cases that continue to progress despite intraocular pressure control suggest that glaucomatous damage is not entirely explained by intraocular pressure. Vascular perfusion insufficiency, mitochondrial dysfunction, oxidative stress, glial cell reactivity, and neuroinflammation are all considered to contribute to RGC vulnerability and optic nerve degeneration (62, 63). Glaucoma therefore provides an important setting for discussing local neuroimmune-metabolic imbalance in primary eye disease.

TSPO evidence in glaucoma comes mainly from RGC pressure injury and neurosteroid protection studies. Ishikawa et al. found in an ex vivo rat high-pressure retinal model that pressure stress can induce retinal TSPO upregulation. The TSPO ligand PK11195 enhanced the synthesis of the neurosteroid allopregnanolone (AlloP) and reduced pressure-induced RGC damage. When 5alpha-reductase inhibitor finasteride blocked AlloP synthesis, the protective effect was attenuated (64). This study suggests that TSPO may improve RGC survival under pressure stress by promoting cholesterol entry into mitochondria and participating in neurosteroid synthesis.

Another line of evidence comes from retinal glial reactivity and ischemic stress. Glaucoma research has shown that microglia, Müller cells, and astrocyte reactivity participate in RGC damage and optic nerve remodeling, but glial reactivity may have dual protective and damaging roles at different stages (65, 66). In a mouse retinal ischemia model, the TSPO ligand XBD173 reduced microglial and Müller cell reactivity and protected inner retinal neurons (22). Although this model is not a glaucoma model, it suggests that TSPO regulation may influence inner retinal glial reactivity under ischemic and inflammatory stress, which has mechanistic relevance to vascular perfusion insufficiency, mitochondrial stress, and RGC vulnerability in glaucoma.

Current evidence supports that TSPO may participate in neurosteroid protection under RGC pressure stress and may regulate inner retinal glial reactivity. However, its cellular sources of expression, disease staging relationships, and therapeutic windows in human glaucoma remain unclear. Future research should combine aqueous humor/serum inflammatory factors, mitochondrial stress markers, OCT-measured RNFL/GCC changes, OCTA perfusion metrics, and visual field progression in glaucoma patients to assess whether TSPO-related signaling can reflect RGC vulnerability, glial activation status, or non-intraocular pressure-dependent progression risk.

## 5 Clinical translation and therapeutic applications of TSPO

### 5.1 TSPO as a biomarker

#### 5.1.1 TSPO as a molecular imaging biomarker (TSPO-PET)

TSPO-PET is the most extensively studied direction in TSPO clinical translation, primarily used for *in vivo* visualization of neuroinflammation and myeloid cell-related tissue responses. The first-generation tracer [11C]PK11195 established the foundation for TSPO neuroinflammation imaging, but its non-specific binding is high, signal-to-noise ratio is low, and quantitative analysis is complex. Second-generation tracers, such as [11C]PBR28, [18F]DPA-714, [18F]GE-180, and [18F]FEDAA1106, generally have higher affinity or superior imaging characteristics, but the binding of some tracers is affected by the TSPO rs6971 polymorphism. In clinical research, genotyping, ligand selection, and standardized quantitative protocols should therefore be combined to reduce the impact of inter-individual binding differences on result interpretation (68).

In systemic or CNS immune diseases, TSPO-PET has shown value in complementing conventional structural imaging. MS studies have shown that TSPO-PET can reveal persistent inflammatory signals at chronic active lesion edges, in normal-appearing white matter and grey matter, helping to assess diffuse innate immune activation that MRI cannot fully reflect (51–53). Autoimmune encephalitis research further suggests that TSPO-PET/MRI can display abnormal uptake in some MRI-negative or MRI-mismatched regions, providing supplementary information for identifying neuroinflammation beyond structural damage (69). In primary CNS vasculitis and some inflammatory vascular diseases, [18F]DPA-714 and other TSPO tracers have also been used to display regional signals associated with vascular wall or brain parenchymal inflammation (56). These findings support TSPO-PET as a cross-disease molecular inflammation readout tool, but its signal does not equate to a single cell type or single inflammatory pathway. It requires interpretation in conjunction with pathological background, MRI/OCT structural imaging, and clinical disease activity.

Compared with CNS diseases, TSPO-PET application in ophthalmic diseases remains at an early stage. The small eye volume, thin retinal and choroidal structures, complex orbital background tissue, and limited clinical PET spatial resolution all constrain direct retinal-level inflammation imaging. TSPO-PET is therefore more suitable as an animal model, pharmacodynamic research, and mechanism validation tool in local eye diseases such as uveitis, DR, and AMD, rather than as a mature clinical detection method (70). For diseases involving the visual pathway or optic nerve degeneration, such as MS-related optic neuritis and glaucoma, brain/visual pathway TSPO-PET may be more practically feasible, and can be used to assess glial reactivity and inflammatory burden in the CNS or optic nerve pathway.

Future TSPO-PET translation in ophthalmology should avoid pursuing the sole technical goal of retinal PET imaging. Instead, it should develop into a multimodal inflammation assessment system. A more feasible strategy is to combine TSPO-PET with PET/MRI, OCT/OCTA, fluorescein or indocyanine green angiography, aqueous humor/vitreous inflammatory factors, and functional assessment, providing molecular-level supplementary evidence for whether systemic immune or metabolic abnormalities have translated into target tissue inflammatory burden.

#### 5.1.2 TSPO as a circulating or fluid biomarker

Compared with TSPO-PET, fluid biomarkers offer advantages of convenient sampling, lower cost, and repeatable monitoring. TSPO can be explored in peripheral immune cells, serum/plasma-related fractions, and intraocular fluid, but its detection form and biological meaning still require standardization. DR patient serum TSPO studies suggest that TSPO and microglia-related markers such as Iba-1 and GLUT5 may reflect early neurovascular abnormalities (9). Glaucoma studies have shown that TSPO expression is elevated in PBMCs of patients with high intraocular pressure, suggesting that peripheral immune cell TSPO may be associated with intraocular neurodegenerative damage (67). These findings provide preliminary support for TSPO as a systemic-ocular crosstalk fluid biomarker.

Future fluid TSPO research should focus on three issues. First, clarifying the detection target: whether it is total TSPO protein, soluble TSPO, exosomal TSPO, or TSPO expression in specific immune cell subpopulations. Second, clarifying the disease stage: TSPO may have different significance in acute inflammation, chronic low-grade inflammation, neurodegenerative stages, and post-treatment repair stages. Third, establishing combined metrics: a single TSPO marker may lack disease specificity and should be combined with cytokines, metabolites, peripheral immune phenotypes, OCT/OCTA parameters, and clinical activity scores for modelling. In uveitis, DR, and AMD in particular, simultaneous detection of aqueous humor or vitreous TSPO and peripheral blood TSPO may reveal the gradient of systemic inflammation transition to intraocular inflammation.

### 5.2 Therapeutic targeting of TSPO

#### 5.2.1 Application of TSPO targeting in systemic diseases

The therapeutic potential of TSPO ligands stems primarily from their regulation of mitochondrial function, neurosteroid synthesis, and inflammatory responses. However, these targeting compounds are pharmacologically heterogeneous. Because no consensus agonist-antagonist classification has been established for TSPO ligands, these compounds are more appropriately distinguished by their chemical scaffold, binding profile, target selectivity, and level of pharmacological development than assigned fixed functional labels.

PK11195, an isoquinoline carboxamide, and Ro5-4864, a benzodiazepine derivative, are classical reference ligands widely used to characterize TSPO expression and function (71). XBD173 (emapunil) is a selective synthetic ligand investigated for its capacity to enhance neurosteroid-associated GABAergic signaling. It can promote neurosteroid generation and produce anti-anxiety effects, with a different mechanism of action compared with traditional benzodiazepine drugs (72). By contrast, etifoxine is a multi-target anxiolytic that directly modulates GABA_A_ receptors while also producing effects attributed to TSPO-associated neurosteroidogenesis (73). PIGA compounds comprise a chemically defined series of high-affinity *N,N*-dialkyl-2-phenylindol-3-ylglyoxylamides that remain predominantly preclinical. Among them, PIGA1138 has been examined for effects on steroidogenesis and astrocyte metabolic fitness (47). DBI-derived peptides represent an endogenous TSPO signaling axis and may mediate macroglia-to-microglia regulation in the retina (8).

Furthermore, the effects of TSPO ligands are significantly context-dependent. Different ligands may exhibit agonist, antagonist, or functionally selective modulation; some effects may also be related to non-TSPO targets (see **Table 3**). In inflammation and neurodegenerative disease models, TSPO ligands can exert protective effects by reducing ROS production, inhibiting the NLRP3 inflammasome, regulating microglial responses, and improving mitochondrial function (47). In BV-2 microglia, PK11195 and midazolam can inhibit LPS/ATP-induced ROS production, NLRP3, caspase-1, and IL-1beta and IL-18 release, and TSPO knockdown can attenuate some drug effects, suggesting that this process is at least partially TSPO-dependent (46, 47). TSPO-targeted therapy in systemic diseases therefore requires more rigorous target validation, dose-response relationship studies, and long-term safety assessment.

**Table 3:** Pharmacological interpretation of representative TSPO ligands.

Group	Agents	Key finding	Study & evidence profile
Classical Reference Ligands	PK11195 Ro5-4864	Nrf2-associated protection in oxidative-stressed ARPE-19 cells Pharmacological comparator in BV-2 microglial and 661W photoreceptor studies Attenuation of Ginkgolide B-induced endothelial protection	Rashid et al., 2020 (35), D4, T1; Feng et al., 2020 (46), D5, T2; Corsi et al., 2022 (37), D4, T1; Lu et al., 2026 (36), D2/3, T1
Selective Neurosteroidogenic Ligands	XBD173 (emapunil)	A promising target to control microglial & retinal reactivity Protective effects in light-induced damage, retinal ischemia, and laser-induced CNV mouse models	Karlstetter et al., 2014 (7), D1/2/4, T2; Scholz et al., 2015 (21), D2, T1; Mages et al., 2019 (22), D2, T1; Wolf et al., 2020 (27), D2/3, T1
Multi-Target Ligands	Etifoxine	Increase cholesterol efflux in RF/6A choroidal endothelial cell stress models Reduce ROS in HFD mouse model of systemic-metabolic and outer retinal injury	Biswas et al., 2018 (19), D4, T1; Biswas et al., 2021 (74), D2, T1
PIGA-Class Ligands	PIGA1138 and derivatives	Modulate inflammatory process in LPS-challenged 661W photoreceptor-like cells Topical eye-drop administration in *rd10* retinal degeneration mice preserved visual function	Corsi et al., 2022 (37), D4, T1; Corsi et al., 2025 (38), D2, T1
Endogenous TSPO-Related Signaling	DBI-derived peptides (e.g., TTN)	Müller glia-to-microglia crosstalk in retinal injury OIR model and glia co-culture systems	Wang et al., 2014 (8), D2/3/4, T2; Gao et al., 2022 (45), D1/2/3, T1
Non-Selective Pharmacological Comparators	Midazolam	Suppression of ROS/NLRP3/cytokine axis in BV-2 cells Partial attenuation of protective effects following TSPO knockdown	Feng et al., 2020 (46), D5, T2

HFD, high fat diet.

#### 5.2.2 Potential application of TSPO targeting in ocular diseases

Ocular diseases present unique opportunities for TSPO-targeted therapy. Compared with systemic administration, local ocular delivery can increase target tissue exposure, reduce systemic side effects, and allow high-frequency efficacy monitoring through OCT, ERG, and fundus imaging methods. In retinal degeneration models, XBD173 can reduce microglial pro-inflammatory responses and protect retinal structure (21). In ischemic retinal injury models, XBD173 can reduce glial reactivity and decrease neuronal death (22). *In vitro* photoreceptor-like cell degeneration models, TSPO targeting can reduce inflammation and apoptosis-related changes, suggesting it may directly or indirectly protect the neuroretina (37).

In DR, potential roles include inhibiting high-glucose-induced mitochondrial ROS, inflammasome activation, and BRB disruption. In AMD, TSPO targeting may simultaneously act on RPE lipid metabolism and microglial inflammation. In glaucoma, TSPO regulation may exert neuroprotective effects by improving RGC mitochondrial stress, reducing pyroptosis/apoptosis, and regulating glial cell inflammation (37, 41, 47, 67). Furthermore, recent research has explored delivering selective TSPO ligands as ophthalmic suspension for retinal degeneration disease models, suggesting that non-invasive ocular delivery may become a future direction (38).

Despite clear prospects, TSPO-targeted therapy for eye diseases remains at the preclinical stage. A key challenge is distinguishing between inhibiting harmful inflammation and preserving protective immune responses. Retinal microglia play roles in debris clearance and homeostasis maintenance in early injury, and excessive inhibition may affect tissue repair. The ideal TSPO therapeutic strategy should therefore be stage-specific and cell-specific immunometabolic remodeling, rather than broad immunosuppression.

### 5.3 Key challenges in ocular TSPO-targeted therapy

#### 5.3.1 Cell-specific targeting

Microglia, RPE, Müller glia, endothelial cells, infiltrating macrophages, retinal neurons, and peripheral immune cells can all express TSPO. TSPO regulation has different functions in different cells: in RPE it may favor cholesterol handling and mitochondrial homeostasis; in microglia it favors inflammation and phagocytosis; in neurons it may involve cell death and energy metabolism; in endothelial cells it may affect barrier function. Future therapeutic design needs to rely on cell-targeted delivery systems, single-cell pharmacodynamic analysis, and conditional genetic models, rather than treating TSPO as a unidirectional pro-inflammatory or anti-inflammatory switch.

#### 5.3.2 Novel drug forms

Existing TSPO ligands are mostly small molecules with good membrane permeability, but they have issues with ligand selectivity, species differences, rs6971-related affinity differences, and potential off-target effects. Three types of novel drug forms can be explored in the future. First, functionally selective TSPO modulators could be developed to preferentially exert anti-inflammatory, mitochondrial-protective, or neurosteroid-regulatory effects. Second, nanocarrier-, liposome-, or micelle-based delivery systems may improve drug targeting to the retina or RPE. Third, strategies that regulate TSPO expression using nucleic acid-based approaches or targeted protein degradation technologies may enable more precise and stage-specific therapeutic intervention.

#### 5.3.3 Local delivery strategies

Ocular drug delivery routes include topical administration, intravitreal injection, suprachoroidal injection, sustained-release implants, and nano-delivery. For anterior segment, topical administration offers advantages in terms of safety, non-invasiveness, and patient convenience, while trans-corneal or trans-scleral delivery may provide additional options for selected ocular targets. However, access to posterior segment tissues through topical routes is limited by ocular anatomical and physiological barriers. For lesions involving the RPE, outer retina, or choroid, suprachoroidal or intravitreal administration may provide more direct and effective drug delivery. For chronic diseases, sustained-release implants can reduce dosing frequency. TSPO ligands are typically lipophilic, and optimizing intraocular distribution, avoiding non-specific accumulation in the lens or optic nerve, and maintaining therapeutic concentrations are key focuses for local delivery research.

#### 5.3.4 Safety and tolerability

TSPO participates in cholesterol transport, steroidogenesis, mitochondrial function, and immune regulation, so long-term intervention may affect normal cellular homeostasis. Intraocular chronic delivery especially requires assessment of RPE phagocytic function, photoreceptor metabolism, vascular homeostasis, immune surveillance, and intraocular pressure changes. Systemic application also requires attention to neuropsychiatric effects, endocrine effects, hepatic and renal metabolism, immunosuppression risk, and drug interactions. Given that TSPO expression regulation differs across species, rodent safety results cannot be simply extrapolated to humans. Non-human primates, humanized cell models, and patient-derived organoids will become important complements.

## 6 Future directions and research frontiers

### 6.1 Development of systemic-ocular dual-disease models

#### 6.1.1 Construction of TSPO-mediated cross-organ disease animal models

Verifying whether TSPO participates in systemic-ocular immune crosstalk requires cross-organ models that can simultaneously capture systemic immune/metabolic abnormalities, ocular barrier changes, and retinal local inflammatory responses. Current model systems remain relatively fragmented: systemic autoimmune or metabolic models typically focus on primary target organs and rarely systematically assess the retina, choroid, and optic nerve, while ocular disease models are mostly based on local injury and struggle to distinguish the relative contributions of systemic inflammatory background and intraocular primary disease. Future model design should therefore not simply superimpose two disease models, but should establish experimental systems with clearly defined question and interpretable controls.

One feasible direction is to construct systemic priming-ocular challenge models. For example, applying mild ocular injury against a background of diabetes, obesity, systemic low-grade inflammation, or autoimmunity to compare whether systemic factors alter BRB permeability, intraocular myeloid cell responses, and neurovascular unit vulnerability. Such studies should include three control groups: systemic model, ocular model, and combined model, with simultaneous detection of peripheral immune phenotype, intraocular inflammatory factors, OCT/angiography phenotype, and TSPO expression, to determine whether TSPO changes originate from peripheral immune priming, local intraocular response, or their interaction.

A second direction is to develop cell-specific Tspo intervention models. Systemic Tspo knockout or drug intervention cannot distinguish the contributions of peripheral immune cells, retinal microglia, Müller glia, RPE, and vascular endothelial cells. Using the Cre-LoxP system to delete or regulate Tspo in specific cell populations can help answer the relative roles of TSPO from different cellular sources in barrier function, myeloid cell activation, lipid metabolism, and neuroprotection. It is particularly important to distinguish TSPO function in resident microglia versus peripheral infiltrating monocytes/macrophages, as the biological implications of the two in systemic-ocular crosstalk may differ.

A third direction is to establish multi-scale readout systems. Cross-organ models should simultaneously integrate molecular, cellular, imaging, and functional endpoints, including peripheral immune cell TSPO, intraocular fluid inflammatory factors, retinal/choroidal tissue TSPO localization, OCT/OCTA or fluorescein angiography, electroretinography, and behavioral visual function. For TSPO-PET, priority should be given to mechanism validation and pharmacodynamic evaluation in animal models, rather than simple analogy to clinical retinal imaging tools. Only by demonstrating temporal relationships between TSPO changes and systemic inflammation, barrier disruption, and intraocular cellular responses in models can further support for TSPO as a systemic-ocular immune crosstalk biomarker or intervention target be established.

#### 6.1.2 Human-relevant organoid and organ-on-chip models

Animal models have advantages in overall physiological environment and immune system integrity, but have limitations in species differences, human-specific mechanisms, and high-throughput drug screening. Human retinal organoids, RPE-endothelial barrier chips, and immune cell co-culture systems provide complementary platforms for dissecting TSPO-related ocular immunometabolic mechanisms. It should be emphasized that these models cannot fully reconstruct systemic-ocular crosstalk, but can be used to decompose key interfaces, such as microglia-neuron interactions, RPE lipid stress, inner and outer BRB barrier function, and immune cell trans-barrier responses.

Retinal organoids with microglia co-culture can be used to simulate interactions between human retinal neural cells and immune cells. In this system, immunometabolic stress can be induced through high glucose, oxidized LDL, complement stimulation, inflammatory factors, or hypoxia, to observe TSPO dynamics in microglia, Müller glia, and neuron-like cells, and to evaluate the effects of TSPO ligands or genetic intervention on glial activation, neuronal survival, and inflammatory factor release.

RPE-choroidal endothelial barrier chips are more suitable for studying the outer BRB and the retinal-choroidal interface. Microfluidic models containing human RPE monolayers and endothelial microvascular structures can assess the effects of inflammation, lipid load, or high glucose on barrier integrity under fluid shear stress and barrier function monitoring conditions. Combining such chips with TSPO intervention can test whether TSPO participates in RPE lipid handling, endothelial inflammation, VEGF release, and barrier permeability changes. A more ideal future platform would incorporate RPE, choroidal endothelium, monocytes/macrophages, and complement components into the same microphysiological system, more closely approximating the pathological interfaces in AMD, DR, and immune-related ocular inflammation.

### 6.2 In-depth analysis of TSPO regulatory mechanisms

#### 6.2.1 Single-cell and spatial multi-omics across cell types and disease stages

TSPO function may differ across eye diseases, cell types, and disease stages. Single-cell and spatial multi-omics can be used to resolve the identity, state, and spatial distribution of TSPO-expressing cells, and are particularly suited to addressing three questions: whether TSPO primarily originates from resident microglia, infiltrating monocytes/macrophages, Müller glia, RPE, or vascular-associated cells; whether TSPO expression shifts from compensatory stress response to pro-inflammatory or pro-degenerative states with disease stage; and whether TSPO-high cells have specific metabolic, inflammatory, or phagocytic programs.

Since TSPO is an outer mitochondrial membrane protein, mRNA levels cannot fully represent its protein expression, subcellular localization, or ligand binding capacity. Future research should therefore integrate single-cell transcriptomics, spatial transcriptomics, spatial proteomics, metabolomics, immunostaining, and *in situ* hybridization validation. The retina has a highly ordered laminar structure, making spatial omics particularly suitable for analyzing the relationship between TSPO expression and the RPE-Bruch membrane-choroid interface, inner retinal vasculature, ganglion cell layer, and inflammatory cell aggregation regions. Through cross-disease and cross-stage comparison, it can be further determined whether TSPO is a generalized cellular stress marker or has an intervention-relevant regulatory role in specific cell states.

#### 6.2.2 Co-regulatory molecules and signaling axes in TSPO-related immune metabolism

TSPO does not function in isolation; rather, it is embedded within a network of mitochondrial, cytoplasmic, and extracellular regulators that collectively shape its context-dependent effects in ocular immunometabolic crosstalk. Key signaling axes identified to date include: the TSPO-NOX1-ROS axis, in which TSPO gates calcium-dependent NOX1 activation to regulate ROS production in retinal phagocytes (27); the TSPO-VDAC-NLRP3 inflammasome axis, in which TSPO indirectly sets the NLRP3 assembly threshold through regulation of mitochondrial ROS release (9, 29); the TSPO-ABCA1 lipid axis, in which TSPO maintains lipid homeostasis through enhancing ABCA1/ABCG1-mediated cholesterol efflux in RPE and choroidal endothelial cells (12, 19); the TSPO-DBI autocrine/paracrine axis, in which retinal Müller glia suppress excessive activation of neighboring microglia through DBI-TSPO signaling (8); and the TSPO-neurosteroidogenesis axis, in which selective TSPO modulation shifts human microglia toward a protective neurosteroidome profile (48).

An important unresolved question is how the cellular environment determines which of these TSPO-associated pathways becomes dominant. We propose that the putative TSPO-associated mitochondrial interactome is state dependent. Local lipid-peroxidation burden, mitochondrial membrane potential, fusion/fission balance, Ca²^+^ homeostasis, and mitophagic competence may dynamically reorganize the spatial proximity or availability of TSPO-associated proteins. Such reconfiguration could bias the dominant downstream program toward NOX1-dependent ROS production, NLRP3 inflammasome activation, lipid trafficking, or neurosteroidogenesis. This model may help explain why TSPO modulation produces divergent effects across different cell types, disease stages, ligand classes, and local immunometabolic environments.

Testing this hypothesis will require approaches capable of distinguishing direct molecular interactions from indirect pathway associations. Proximity-labelling proteomics, cross-linking, co-immunoprecipitation, and live-cell imaging, combined with cell-specific genetic perturbation, could help define the TSPO-associated interactome. These studies should be performed under conditions that modulate lipid peroxidation, Ca²^+^ flux, and mitochondrial dynamics. Time-resolved multi-omics profiling may further clarify whether interactome changes precede or follow activation of downstream inflammatory, metabolic, and redox-related pathways.

The integration of these mechanistic studies with single-cell and spatial multi-omics, TSPO-PET, OCT/OCTA, fluid biomarkers, and longitudinal clinical outcomes will generate high-dimensional and heterogeneous datasets that cannot be adequately interpreted using conventional univariate analyses alone. Systems-biology approaches could integrate multi-omic and imaging data to infer cell-state-specific TSPO regulatory networks and identify the dominant molecular pathways linking TSPO activity to ocular phenotypes. Multimodal machine-learning models could then use these network-derived features to stratify patients and predict individual responses to TSPO modulators.

## 7 Conclusion

This review proposes a three-stage framework of TSPO-mediated systemic-ocular immunometabolic crosstalk, integrating peripheral immunometabolic priming, BRB vulnerability transition, and intraocular glial amplification into a coherent mechanistic narrative. Across systemic autoimmune diseases, diabetic retinopathy, and primary ocular diseases with systemic components, TSPO evidence converges on mitochondrial stress sensing, lipid homeostasis, redox regulation, and immune cell polarization as the shared mechanistic threads. Local, functionally selective TSPO modulation may become therapeutically useful, but clinical translation depends on causal ocular evidence, cell-specific manipulation, and paired systemic-ocular longitudinal biomarker studies.
