# Supplementary material for: TSPO in systemic-ocular immunometabolic crosstalk: mechanisms and therapeutic implications
Source: Front Immunol. 2026 Oct 6;17:1929816. doi: 10.3389/fimmu.2026.1929816 (PMC13681183; doi:10.3389/fimmu.2026.1929816)
Supplement: Supplementary file 1 [file Supplementaryfile1.docx]

**Supplementary Methods**

*TSPO in Systemic-Ocular Immunometabolic Crosstalk: Mechanisms and Therapeutic Implications*

S1. Review scope and literature identification

This narrative review was informed by structured literature searches conducted in PubMed and Embase from database inception through August 2026. The searches were designed to identify complementary evidence relevant to TSPO biology and systemic–ocular immunometabolic crosstalk, including ocular studies, systemic immunometabolic studies, mechanistic investigations, and ligand- or tracer-specific studies. Evidence from human studies, animal models, ex vivo tissues, primary cells, and established cell lines was considered where it contributed to the mechanistic, disease-related, biomarker, or pharmacological synthesis. Detailed database-specific search strategies are provided below.

S2. Database search strategy

Searches were organized into four complementary domains. The ocular search identified direct ocular TSPO evidence; the systemic immunometabolic search captured evidence relevant to peripheral immune and metabolic priming; mechanistic searches addressed mitochondrial, redox, inflammasome, lipid, and cell-state pathways; and ligand-specific searches supported the pharmacological synthesis. Search terms were adapted to database syntax. No pooled meta-analysis was undertaken.

S2.1 Ocular TSPO evidence

**PubMed:** (TSPO[Title/Abstract] OR "translocator protein 18 kDa"[Title/Abstract] OR "peripheral benzodiazepine receptor"[Title/Abstract]) AND (eye[Title/Abstract] OR ocular[Title/Abstract] OR retina*[Title/Abstract] OR "retinal pigment epithelium"[Title/Abstract] OR RPE[Title/Abstract] OR choroid*[Title/Abstract] OR "blood-retinal barrier"[Title/Abstract] OR "blood aqueous barrier"[Title/Abstract] OR "optic nerve"[Title/Abstract] OR cornea*[Title/Abstract] OR conjunctiv*[Title/Abstract] OR sclera*[Title/Abstract] OR uvea*[Title/Abstract] OR iris[Title/Abstract] OR "ciliary body"[Title/Abstract] OR lacrimal[Title/Abstract] OR orbit*[Title/Abstract] OR glaucoma*[Title/Abstract] OR "diabetic retinopathy"[Title/Abstract] OR "age-related macular degeneration"[Title/Abstract] OR AMD[Title/Abstract] OR uveitis[Title/Abstract])

**Embase:** (tspo OR 'translocator protein 18 kda' OR 'peripheral benzodiazepine receptor'):ti,ab AND (eye OR ocular OR retina* OR 'retinal pigment epithelium' OR rpe OR choroid* OR 'blood-retinal barrier' OR 'blood aqueous barrier' OR 'optic nerve' OR cornea* OR conjunctiv* OR sclera* OR uvea* OR iris OR 'ciliary body' OR lacrimal OR orbit* OR glaucoma* OR 'diabetic retinopathy' OR 'age-related macular degeneration' OR amd OR uveitis):ti,ab

S2.2 Systemic immunometabolic TSPO evidence

**PubMed:** (TSPO[Title/Abstract] OR "translocator protein 18 kDa"[Title/Abstract] OR "peripheral benzodiazepine receptor"[Title/Abstract]) AND (immune*[Title/Abstract] OR inflamm*[Title/Abstract] OR immunometab*[Title/Abstract] OR monocyte*[Title/Abstract] OR macrophage*[Title/Abstract] OR "peripheral blood mononuclear cell*"[Title/Abstract] OR PBMC*[Title/Abstract] OR inflammasome*[Title/Abstract] OR NLRP3[Title/Abstract] OR "metabolic stress"[Title/Abstract] OR diabetes[Title/Abstract] OR diabetic[Title/Abstract] OR obesity[Title/Abstract] OR hyperlipid*[Title/Abstract] OR dyslipid*[Title/Abstract] OR cholesterol[Title/Abstract] OR lipid*[Title/Abstract] OR autoimmune*[Title/Abstract] OR "multiple sclerosis"[Title/Abstract] OR vasculitis[Title/Abstract] OR sepsis[Title/Abstract] OR sarcoidosis[Title/Abstract])

**Embase:** (tspo OR 'translocator protein 18 kda' OR 'peripheral benzodiazepine receptor'):ti,ab AND (immune* OR inflamm* OR immunometab* OR monocyte* OR macrophage* OR 'peripheral blood mononuclear cell*' OR pbmc* OR inflammasome* OR nlrp3 OR 'metabolic stress' OR diabetes OR diabetic OR obesity OR hyperlipid* OR dyslipid* OR cholesterol OR lipid* OR autoimmune* OR 'multiple sclerosis' OR vasculitis OR sepsis OR sarcoidosis):ti,ab

S2.3 Mechanistic TSPO evidence

**PubMed:** (TSPO[Title/Abstract] OR "translocator protein 18 kDa"[Title/Abstract] OR "peripheral benzodiazepine receptor"[Title/Abstract]) AND (mitochondri*[Title/Abstract] OR calcium[Title/Abstract] OR "reactive oxygen species"[Title/Abstract] OR ROS[Title/Abstract] OR NLRP3[Title/Abstract] OR VDAC[Title/Abstract] OR NOX1[Title/Abstract] OR cholesterol[Title/Abstract] OR lipid*[Title/Abstract] OR neurosteroid*[Title/Abstract] OR ferroptosis[Title/Abstract] OR autophagy[Title/Abstract] OR mitophagy[Title/Abstract] OR bioenergetic*[Title/Abstract] OR PPAR*[Title/Abstract])

**Embase:** (tspo OR 'translocator protein 18 kda' OR 'peripheral benzodiazepine receptor'):ti,ab AND (mitochondri* OR calcium OR 'reactive oxygen species' OR ros OR nlrp3 OR vdac OR nox1 OR cholesterol OR lipid* OR neurosteroid* OR ferroptosis OR autophagy OR mitophagy OR bioenergetic* OR ppar*):ti,ab

S2.4 Ligand- and tracer-specific TSPO evidence

**PubMed:** (TSPO[Title/Abstract] OR "translocator protein 18 kDa"[Title/Abstract] OR "peripheral benzodiazepine receptor"[Title/Abstract]) AND (PK11195[Title/Abstract] OR Ro5-4864[Title/Abstract] OR Ro54864[Title/Abstract] OR XBD173[Title/Abstract] OR emapunil[Title/Abstract] OR etifoxine[Title/Abstract] OR PIGA1138[Title/Abstract] OR PIGA[Title/Abstract] OR "diazepam binding inhibitor"[Title/Abstract] OR DBI[Title/Abstract] OR TTN[Title/Abstract] OR midazolam[Title/Abstract] OR DPA-714[Title/Abstract] OR PBR28[Title/Abstract] OR GE-180[Title/Abstract] OR FEDAA1106[Title/Abstract])

**Embase:** (tspo OR 'translocator protein 18 kda' OR 'peripheral benzodiazepine receptor'):ti,ab AND (pk11195 OR ro5-4864 OR ro54864 OR xbd173 OR emapunil OR etifoxine OR piga1138 OR piga OR 'diazepam binding inhibitor' OR dbi OR ttn OR midazolam OR dpa-714 OR pbr28 OR ge-180 OR fedaa1106):ti,ab

S3. Study prioritization and scope boundaries

Peer-reviewed original studies were prioritized when they directly measured, manipulated, or imaged TSPO in human ocular samples, ocular imaging cohorts, ocular animal models, ex vivo ocular tissues, primary ocular cells, organoids, or ocular cell lines. Human systemic, CNS, and nonocular mechanistic studies were retained when they directly informed peripheral immunometabolic priming, TSPO target attribution, ligand pharmacology, or a mechanistic link used in the proposed systemic–ocular framework. Reviews, guidelines, and general disease-background references were used for contextual synthesis but were not assigned study-level D/T evidence codes.

The literature search was not restricted to any specific ocular compartment, and the synthesis was organized according to the mechanistic and translational relevance of the available evidence rather than as a compartment-by-compartment anatomical survey of the eye. Accordingly, differences in the depth of coverage across ocular compartments reflect the uneven distribution and maturity of the current evidence base rather than an a priori anatomical restriction. Extrapolation of the proposed framework to less-studied ocular compartments should therefore be approached with caution.

S4. Structured evidence appraisal

The evidence base includes human observational studies, PET and OCT-linked biomarker studies, in vivo disease models, ex vivo tissues, primary cells, immortalized cell lines, and ligand-based pharmacological experiments. Because these designs address heterogeneous mechanistic questions rather than a common intervention-outcome pair, evidence was not collapsed into a single GRADE-style certainty rating. Instead, each primary study directly informing the mechanistic, systemic-ocular, or pharmacological synthesis was characterized along two descriptive dimensions: biological directness to the ocular question (D1-D5) and strength of TSPO attribution (T0-T3). Multiple D codes were permitted when a single study integrated more than one biological level.

| **Dimension** | **Code** | **Definition** |
| --- | --- | --- |
| Biological directness | D1 | Human ocular tissue, ocular fluid, ocular imaging, or directly relevant clinical ocular evidence. |
| Biological directness | D2 | In vivo ocular disease model. |
| Biological directness | D3 | Ex vivo ocular tissue, primary ocular cells, or organoid. |
| Biological directness | D4 | Immortalized or commercial ocular cell line. |
| Biological directness | D5 | Nonocular or systemic model used to inform the ocular framework. |
| TSPO attribution | T3 | Cell-specific genetic manipulation with rescue, or convergent genetic and pharmacological validation supporting TSPO dependence. |
| TSPO attribution | T2 | Global knockout/knockdown, or pharmacological evidence with explicit target-engagement, competition, or genetic attenuation of the pharmacological effect. |
| TSPO attribution | T1 | Ligand-only experiment, TSPO expression/binding association, or imaging signal without direct target validation. |
| TSPO attribution | T0 | Indirect mechanistic or pharmacological extrapolation without direct TSPO validation in the experimental system. |

S5. Interpretation and reporting of evidence profiles

The D and T dimensions are complementary descriptors rather than an overall methodological-quality score. A study can be biologically direct but causally weak (for example, a small cross-sectional human biomarker study), or mechanistically strong but biologically indirect to the eye (for example, a nonocular genetic model). Ligand-only findings were interpreted cautiously because TSPO ligands can exhibit context-dependent, ligand-specific, and off-target effects. Likewise, TSPO-PET signal was treated as a molecular imaging readout rather than as proof of a unique cell type or single inflammatory pathway.
